# Supplementary material for: Exploring Potentilla nepalensis Phytoconstituents: Integrated Strategies of Network Pharmacology, Molecular Docking, Dynamic Simulations, and MMGBSA Analysis for Cancer Therapeutic Targets Discovery
Source: Pharmaceuticals (Basel). 2024 Jan 19;17(1):134. doi: 10.3390/ph17010134 (PMC10819299; doi:10.3390/ph17010134)

Figure S3. Root n-hexane extract PCs enrichment analysis, A. Biological process, B. Molecular function, C. Cellular components, D. Pathways, E. Diseases, F. Drugs

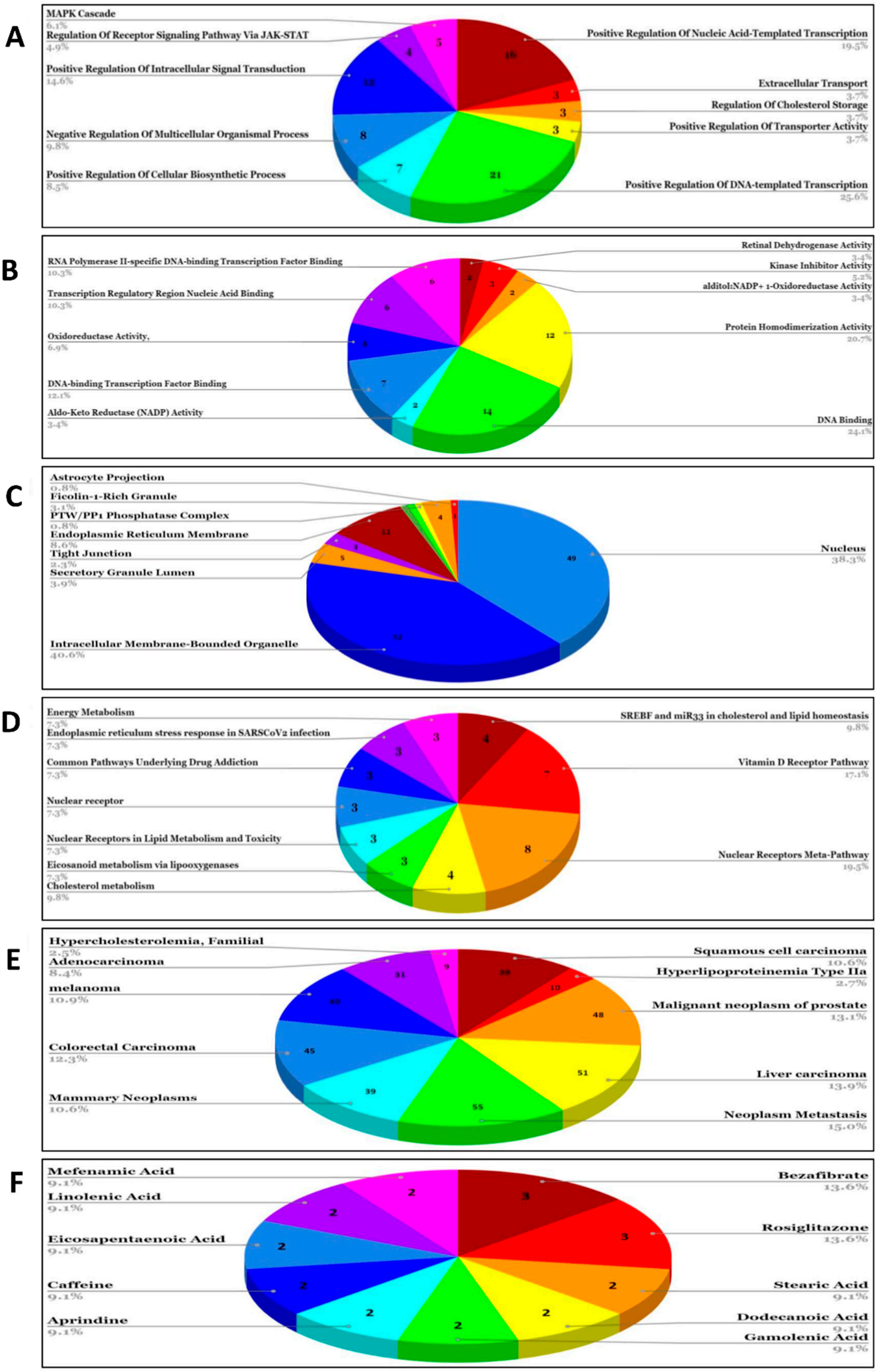

Supplement: Supplementary file 1 [file pharmaceuticals-17-00134-s001.zip › Figure S3.pdf]
