# Supplementary material for: Exploring Potentilla nepalensis Phytoconstituents: Integrated Strategies of Network Pharmacology, Molecular Docking, Dynamic Simulations, and MMGBSA Analysis for Cancer Therapeutic Targets Discovery
Source: Pharmaceuticals (Basel). 2024 Jan 19;17(1):134. doi: 10.3390/ph17010134 (PMC10819299; doi:10.3390/ph17010134)
Supplement: Supplementary file 1 [file pharmaceuticals-17-00134-s001.zip › Table S1.pdf]

Table S1. Major phytocompounds identified in GC-MS profiling of methanolic extracts of roots (MR) of *P. nepalensis*

Sl. No	Compounds	Chemical formula	SMILES format	PubChem ID	2D Structures
1a	Tetradecanoic acid, 10,13-dimethyl-, methyl ester	$C_{17}H_3O_4$	CC(C)CCC(C)CCCCCCCCC(=O)OC	554145	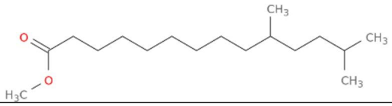
1b	Heptadecanoic acid, 16-methyl-, methyl ester	$C_{19}H_{38}O_2$	CC(C)CCCCCCCCCCCCCCC(=O)OC	110444	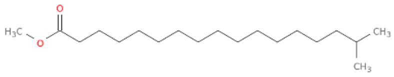
1c	1,1,1,5,7,7,7-Heptamethyl-3,3-bis(trimethylsiloxy) tetrasiloxane	$C_{13}H_{40}O_5Si_6$	C[Si](O[Si](C)(C)C)O[Si](O[Si](C)(C)C)(O[Si](C)(C)C)O[Si](C)(C)C	6329081	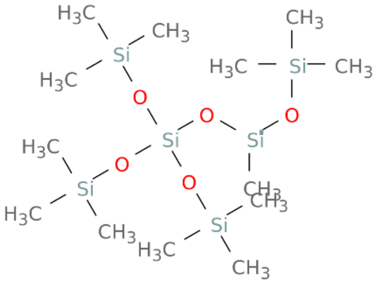
1d	Phthalic acid, butyl hept-4-yl	$C_{19}H_{28}O_4$	CCCCOC(=O)C1=CC=CC=C1C(=O)OC(CCC)CCC	91720764	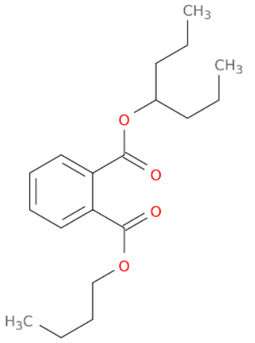
1e	1,1,1,3,5,5,7,7,7-Nonamethyl-3-(trimethyl siloxy) tetrasiloxane	$C_{12}H_{36}O_4Si_5$	C[Si](C)(C)O[Si](C)(C)O[Si](C)(C)O[Si](C)(C)O[Si](C)(C)C	8853	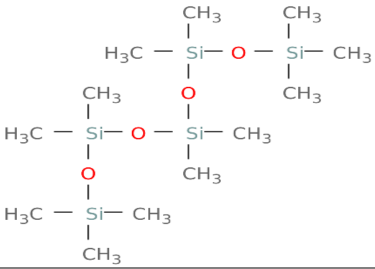
1f	Norepinephrine, (R)-, 4TMS derivative	$C_{20}H_{43}NO_3Si_4$	CCOC1=CC=CC=C1O[C@@H]([C@H]2CNCCO2)C3=CC=CC=C3	12751	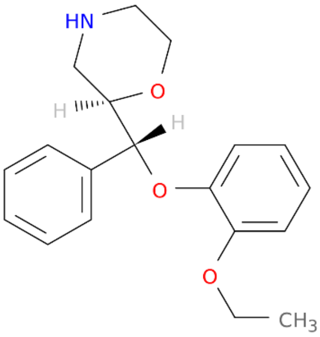
1g	2-Isopropyl-6-phenylnicotinonitrile	$C_{15}H_{14}N_2$	CC(C)C1=C(C=CC(=N1)C2=CC=CC=C2)C#N	610213	

					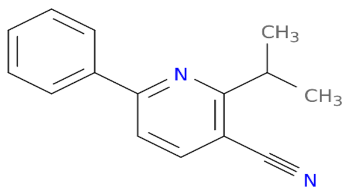
1h	Cyclononasiloxane, octadecamethyl	$C_{18}H_{54}O_9Si_9$	C[Si]1(O[Si](O[Si](O[Si](O[Si](O[Si](O[Si](O[Si](O[Si](O1)(C)C)(C)C)(C)C)(C)C)(C)C)(C)C)(C)C)(C)C)C	11172	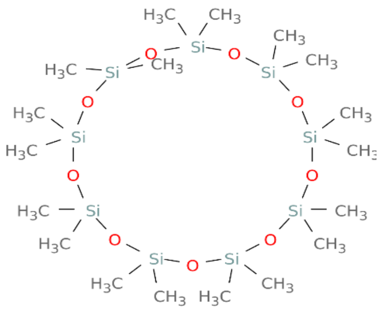
1i	Oxalic acid, 2TMS derivative	$C_8H_{18}O_4Si_2$	C[Si](C)(C)OC(=O)C(=O)O[Si](C)(C)C	519543	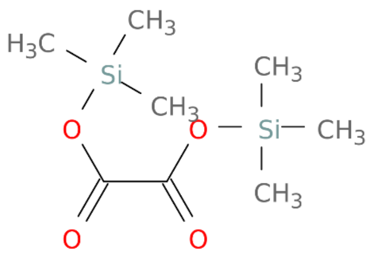
1j	2-Tetradecanol	$C_{14}H_{30}O$	CCCCCCCCCCCCC(C)O	20831	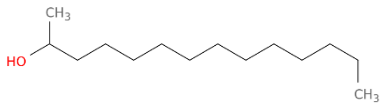
