# Supplementary material for: Exploring Potentilla nepalensis Phytoconstituents: Integrated Strategies of Network Pharmacology, Molecular Docking, Dynamic Simulations, and MMGBSA Analysis for Cancer Therapeutic Targets Discovery
Source: Pharmaceuticals (Basel). 2024 Jan 19;17(1):134. doi: 10.3390/ph17010134 (PMC10819299; doi:10.3390/ph17010134)
Supplement: Supplementary file 1 [file pharmaceuticals-17-00134-s001.zip › Table S2.pdf]

Table S2. Major phytocompounds identified in GC-MS profiling of methanolic extracts of shoots (MS) of *P. nepalensis*.

Sl. No	Compounds	Chemical formula	SMILES format	PubChem ID	2D Structures
2a	Hexadecanoic acid,methyl ester	$C_{17}H_{34}O_2$	CCCCCCCCCCCCCCCC(=O)OC	8181	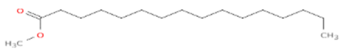
2b	1,1,1,5,7,7,7-Heptamethyl-3,3-bis(trimethylsiloxy)tetrasiloxane	$C_{13}H_{40}O_5Si_6$	C[Si](O[Si](C)(C)C)O[Si](O[Si](C)(C)C)(O[Si](C)(C)C)O[Si](C)(C)C	6329081	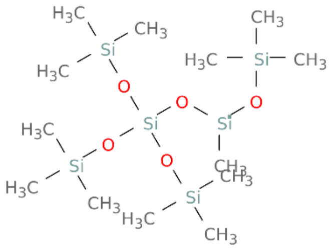
2c	Oxalic acid, 2TMS derivative	$C_8H_{18}O_4Si_2$	C[Si](C)(C)OC(=O)C(=O)O[Si](C)(C)C	519543	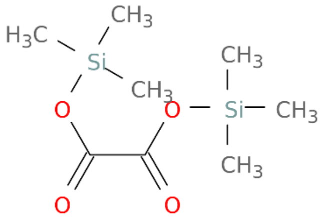
2d	Cyclooctasiloxane, hexadecamethyl-	$C_{16}H_{48}O_8Si_8$	C[Si]1(O[Si](O[Si](O[Si](O[Si](O[Si](O[Si](O[Si](O1)(C)C)(C)C)(C)C)(C)C)(C)C)(C)C)(C)C)C	11170	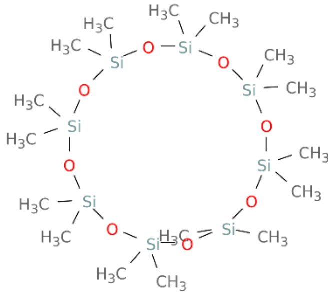
2e	3-Ethyl-7-hydroxyphthalide	$C_{10}H_{10}O_3$	CCC1C2=C(C(=CC=C2)O)C(=O)O1	591119	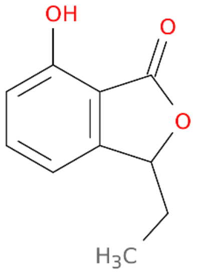
2f	Phthalic acid, butyl hex-3-yl ester	$C_{18}H_{26}O_4$	CCCCOC(=O)C1=CC=CC=C1C(=O)OC(CC)CCC	91720282	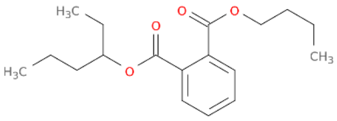
2g	1,1,1,3,5,5,7,7,7-Nonamethyl-3-(trimethylsiloxy)tetrasiloxane	$C_{12}H_{36}O_4Si_5$	C[Si](C)(C)O[Si](C)(C)O[Si](C)(O[Si](C)(C)C)O[Si](C)(C)C	553706	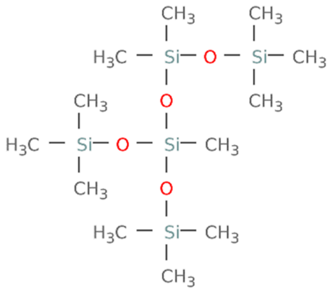

2h	1-Hexyl-1-nitrocyclohexane	$C_{12}H_{23}NO_2$	CCCCCCC1(CCCCC1)[N+](=O)[O-]	544063	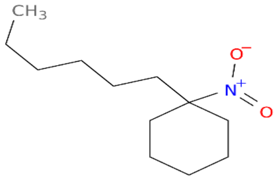
2i	Isobutyl 4-hydroxybenzoate	$C_{11}H_{14}O_3$	CC(C)COC(=O)C1=CC=C(C=C1)O	20240	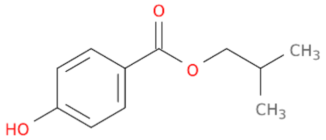
2j	Octanoic acid, 5-(acetyloxy)-, methyl ester	$C_{11}H_{20}O_4$	CCCC(CCCC(=O)OC)OC(=O)C	539496	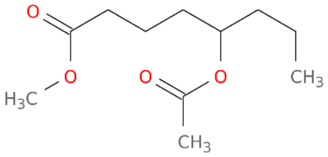
