# Supplementary material for: Exploring Potentilla nepalensis Phytoconstituents: Integrated Strategies of Network Pharmacology, Molecular Docking, Dynamic Simulations, and MMGBSA Analysis for Cancer Therapeutic Targets Discovery
Source: Pharmaceuticals (Basel). 2024 Jan 19;17(1):134. doi: 10.3390/ph17010134 (PMC10819299; doi:10.3390/ph17010134)
Supplement: Supplementary file 1 [file pharmaceuticals-17-00134-s001.zip › Table S3.pdf]

Table S3. Major phytocompounds identified in GC-MS profiling of n-hexane extracts of roots (NR)of *P. nepalensis*.

Sl. No	Compounds	Chemical formula	SMILES format	PubChem ID	2D Structures
3a	Trichloromethyl 9-anthracenecarbodithioate	$C_{16}H_9Cl_3S_2$	C1=CC=C2C(=C1)C=C3C=CC=CC3=C2C(=S)SC(Cl)(Cl)Cl	613595	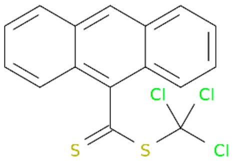
3b	Heptane, 3,3-dimethyl-	C_9H_{20}	CCCCC(C)(C)CC	520991	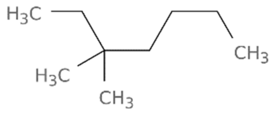
3c	Hexadecane	$C_{16}H_{34}$	CCCCCCCCCCCCCCCC	11006	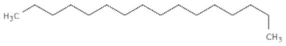
3d	1,1,1,5,7,7,7-Heptamethyl-3, 3-bis(trimethylsiloxy)tetrasiloxane	$C_{13}H_{40}O_5Si_6$	C[Si](O[Si](C)(C)C)O[Si](O[Si](C)(C)C)(O[Si](C)(C)C)O[Si](C)(C)C	6329081	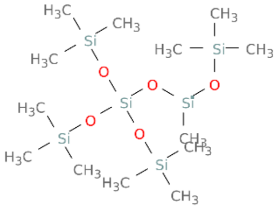
3e	Dodecane	$C_{12}H_{26}$	CCCCCCCCCCCC	8182	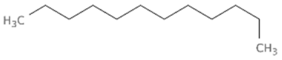
3f	Eicosane	$C_{20}H_{42}$	CCCCCCCCCCCCCCCCCCCC	8222	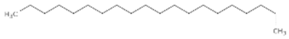
3g	1,1,1,3,5,5,7,7,7-Nonamethyl-3-(trimethylsiloxy) tetrasiloxane Propanoicacid,	$C_{12}H_{36}O_4Si_5$	C[Si](C)(C)O[Si](C)(C)O[Si](C)(C)O[Si](C)(C)O[Si](C)(C)C	8853	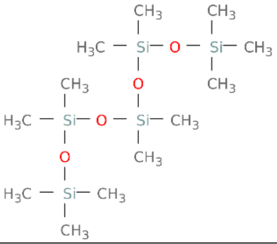
3h	2-oxo-3-(trimethylsilyl)-, trimethylsilyl ester	$C_9H_{20}O_3Si_2$	CCOC(=O)C1(CC2C(=C(C(=O)O2)[Si](C)(C)C)C1)C(=O)OCC	102119459	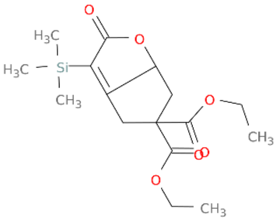
3i	1,1,1,3,5,7,7,7-Octamethyl-3,5-bis(trimethylsiloxy) tetrasiloxane	$C_{14}H_{42}O_5Si_6$	C[Si](C)(C)O[Si](C)(O[Si](C)(C)C)O[Si](C)(O[Si](C)(C)C)O[Si](C)(C)C	553110	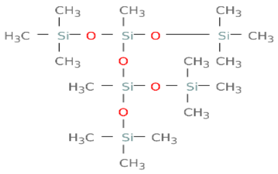
3j	2,4,6,9-Dehydroadamantane	$C_{10}H_{12}$	C1C2CC3C4C1C5C2C5C34	562325	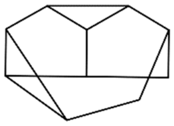
