# Supplementary material for: Exploring Potentilla nepalensis Phytoconstituents: Integrated Strategies of Network Pharmacology, Molecular Docking, Dynamic Simulations, and MMGBSA Analysis for Cancer Therapeutic Targets Discovery
Source: Pharmaceuticals (Basel). 2024 Jan 19;17(1):134. doi: 10.3390/ph17010134 (PMC10819299; doi:10.3390/ph17010134)
Supplement: Supplementary file 1 [file pharmaceuticals-17-00134-s001.zip › Table S4.pdf]

Table S4. Major phytocompounds identified in GC-MS profiling of n-hexane extracts of shoots (NS) of *P. nepalensis*

Sl. No	Compounds	Chemical formula	SMILES format	PubChem ID	2D Structures
4a	Benzene, 1,3,5-tri-tert-butyl-	$C_{18}H_{30}$	CC(C)(C)C1=CC(=CC(=C1)C(C)(C)C)C(C)(C)C	15089	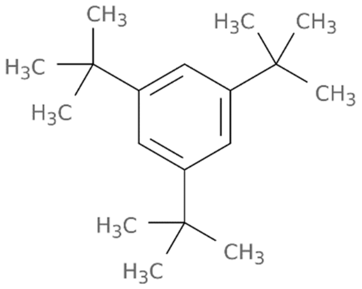
4b	1,1,1,3,5,5,5-Heptamethyltrisiloxane 4H-1-Benzopyran-2-carboxylic acid	$C_7H_{22}O_2Si_3$	C[Si](O[Si](C)(C)C)O[Si](C)(C)C	6327366	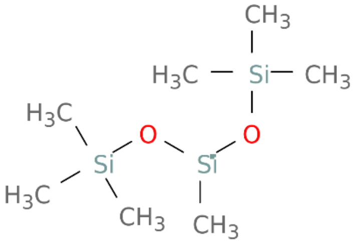
4c	5-amino-6-hydroxy-4-oxo-, ethyl ester	$C_{12}H_{11}NO_5$	CCOC(=O)C1=CC(=O)C2=C(O1)C=CC(=C2N)O	619354	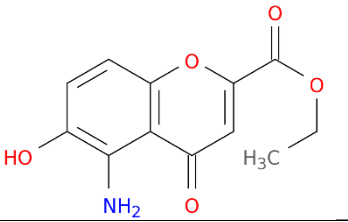
4d	1-Propene, 3-methoxy-	C_4H_8O	COCC=C	69392	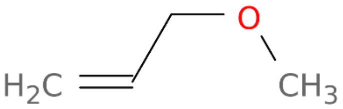
4e	Phthalic acid, butyl hept-4-yl ester	$C_{19}H_{28}O_4$	CCCCOC(=O)C1=CC=CC=C1C(=O)OC(CCC)CCC	91720764	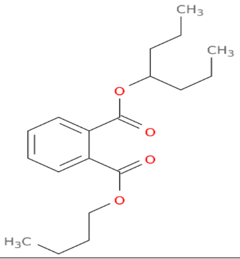
4f	Heptane,	C_9H_{20}	CCCCCCC	8900	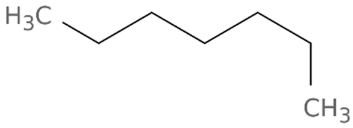
4g	3,3-dimethyl- N-(Methyl sulfonyl)-N, O-bis (trimethyl silyl) hydroxylamine	$C_7H_{21}NO_3SSi_2$	CN([Si](C)(C)C)S(=O)(=O)O[Si](C)(C)C	155620124	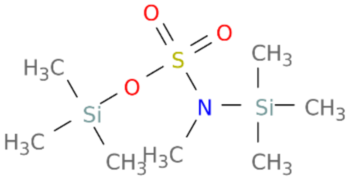
4h	Dodecanoic acid, 2-methyl-	$C_{13}H_{26}O_2$	CCCCCCCCCC[C@@H](C)C(=O)O	5312339	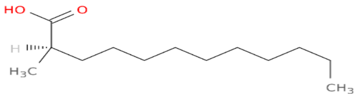

4i	2-Acetyl-3-ethylpyrazine	$C_8H_{10}N_2O$	CCC1=NC=CN=C1C(=O)C	61918	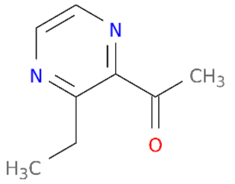
4j	Eicosane	$C_{20}H_{42}$	CCCCCCCCCCCCCCCCCCCC	8222	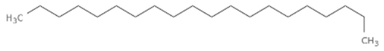
