# Supplementary material for: Exploring Potentilla nepalensis Phytoconstituents: Integrated Strategies of Network Pharmacology, Molecular Docking, Dynamic Simulations, and MMGBSA Analysis for Cancer Therapeutic Targets Discovery
Source: Pharmaceuticals (Basel). 2024 Jan 19;17(1):134. doi: 10.3390/ph17010134 (PMC10819299; doi:10.3390/ph17010134)
Supplement: Supplementary file 1 [file pharmaceuticals-17-00134-s001.zip › Table S5.pdf]

Table S5. The binding affinities (B.A.) *kcal/mol* values of TP53, NFKB1, and HSPCB with the PCs of of *p.nepalensis* (1a – 4j)

TP53							
PCs	B.A.	PCs	B.A.	PCs	B.A.	PCs	B.A.
1a	-6.1	2a	***-8.0**	3a	-7.5	4a	-6.4
1b	***-8.6**	2b	-6.3	3b	-5.2	4b	-6.1
1c	-6.4	2c	-5.6	3c	-7.2	4c	-7.0
1d	-5.0	2d	-3.9	3d	-7.0	4d	-3.8
1e	-6.9	2e	-6.2	3e	-7.3	4e	-6.0
1f	-6.2	2f	-5.8	3f	-4.2	4f	-5.6
1g	-5.8	2g	-6.4	3g	-6.7	4g	-5.4
1h	-7.2	2h	-5.9	3h	-6.3	4h	-5.9
1i	-6.1	2i	-7.1	3i	-5.9	4i	-4.9
1j	-7.8	2j	-5.6	3j	-5.6	4j	-3.9
NFKB1							
1a	-5.2	2a	-5.0	3a	-6.8	4a	-5.3
1b	-4.9	2b	-4.4	3b	-4.3	4b	-5.0
1c	-4.9	2c	-4.9	3c	-3.8	4c	-6.4
1d	-4.9	2d	-4.9	3d	-4.9	4d	-4.9
1e	-4.9	2e	-6.5	3e	-4.3	4e	-4.9
1f	-5.0	2f	-5.1	3f	-3.8	4f	-3.8
1g	-4.4	2g	-4.9	3g	-4.9	4g	-5.0
1h	-5.0	2h	-5.0	3h	-4.4	4h	-4.4
1i	-4.4	2i	-5.5	3i	-5.0	4i	-4.9
1j	-4.0	2j	-4.9	3j	-4.3	4j	-4.9
HSPCB							
1a	-6.3	2a	-5.7	3a	***-9.6**	4a	***-8.7**
1b	-6.3	2b	-6.2	3b	-5.3	4b	-5.6
1c	-6.1	2c	-5.2	3c	-6.3	4c	***-8.2**
1d	-7.5	2d	-7.4	3d	-5.3	4d	-3.4
1e	-7.2	2e	-7.5	3e	-5.9	4e	-7.8
1f	-6.8	2f	-7.7	3f	-6.1	4f	-4.5
1g	-5.8	2g	-6.2	3g	-5.8	4g	-6.8
1h	-3.4	2h	-4.5	3h	-6.6	4h	-6.4
1i	-5.6	2i	-6.6	3i	-7.4	4i	-5.8
1j	-6.4	2j	-6.1	3j	-5.9	4j	-5.8

*Higher binding score exhibited ligands
