# Supplementary material for: Exploring Potentilla nepalensis Phytoconstituents: Integrated Strategies of Network Pharmacology, Molecular Docking, Dynamic Simulations, and MMGBSA Analysis for Cancer Therapeutic Targets Discovery
Source: Pharmaceuticals (Basel). 2024 Jan 19;17(1):134. doi: 10.3390/ph17010134 (PMC10819299; doi:10.3390/ph17010134)
Supplement: Supplementary file 1 [file pharmaceuticals-17-00134-s001.zip › Table S6.pdf]

Table S6. Results from t-test analysis for p53 complexes.

T-test	
Unpaired t test	
P value	<0.0001
P value summary	****
Significantly different (P < 0.05)?	Yes
One- or two-tailed P value?	Two-tailed
t, df	t=139.0, df=3600
Mean of column A p53 + 1b	-47.37
Mean of column B p53 + 2a	-27.3
Difference between means (B - A) ± SEM	20.06 ± 0.1443
95% confidence interval	19.78 to 20.34
R squared (eta squared)	0.8429
F, DFn, Dfd	1.859, 1800, 1800
P value	<0.0001
P value summary	****
Significantly different (P < 0.05)?	Yes
Sample size, column A	1801
Sample size, column B	1801
