# Supplementary material for: Exploring Potentilla nepalensis Phytoconstituents: Integrated Strategies of Network Pharmacology, Molecular Docking, Dynamic Simulations, and MMGBSA Analysis for Cancer Therapeutic Targets Discovery
Source: Pharmaceuticals (Basel). 2024 Jan 19;17(1):134. doi: 10.3390/ph17010134 (PMC10819299; doi:10.3390/ph17010134)
Supplement: Supplementary file 1 [file pharmaceuticals-17-00134-s001.zip › Table S7.pdf]

Table S7. Results from One-way ANOVA test for HSPCB complexes.

One way Anova					
Tukey's multiple comparisons test	Mean Difference	95.00% CI of Difference	Significant	Summary	Adjusted P-Value
HSPCB + 3a vs. HSPCB + 4a	-1.407	-1.574 to -1.239	Yes	****	<0.0001
HSPCB + 3a vs. HSPCB + 4c	-1.543	-1.710 to -1.376	Yes	****	<0.0001
HSPCB + 4a vs. HSPCB + 4c	-0.1366	-0.3038 to 0.03071	No	ns	0.1348

ns. Non-significant.
